# Mass Spectrometry-Based Metabolomics Reveals a Salivary Signature for Low-Severity COVID-19

**DOI:** 10.3390/ijms252211899

**Published:** 2024-11-06

**Authors:** Iasmim Lopes de Lima, Alex Ap. Rosini Silva, Carlos Brites, Natália Angelo da Silva Miyaguti, Felipe Raposo Passos Mansoldo, Sara Vaz Nunes, Pedro Henrique Godoy Sanches, Thais Regiani Cataldi, Caroline Pais de Carvalho, Adriano Reis da Silva, Jonas Ribeiro da Rosa, Mariana Magalhães Borges, Wellisson Vilarindo Oliveira, Thiago Cruz Canevari, Alane Beatriz Vermelho, Marcos Nogueira Eberlin, Andreia M. Porcari

**Affiliations:** 1 PPGEMN, School of Engineering, Mackenzie Presbyterian University, São Paulo 01302-907, SP, Brazil; iasmim.lima@mackenzista.com.br (I.L.d.L.); carolinepais.carvalho1@mackenzista.com.br (C.P.d.C.); adrianoreisjose.silva@mackenzista.com.br (A.R.d.S.); mariana.borges@mackenzista.com.br (M.M.B.); thiago.canevari@mackenzie.br (T.C.C.); 2 MackGraphe—Mackenzie Institute for Research in Graphene and Nanotechnologies, Mackenzie Presbyterian Institute, São Paulo 01302-907, SP, Brazil; 3 MS4Life Laboratory of Mass Spectrometry, Health Sciences Postgraduate Program, São Francisco University—USF, Bragança Paulista 12916-900, SP, Brazil; alex.rosini@mail.usf.edu.br (A.A.R.S.); namiyaguti@gmail.com (N.A.d.S.M.); pedrohgodoys@gmail.com (P.H.G.S.); jonas.rosa@mail.usf.edu.br (J.R.d.R.); andreia.porcari@usf.edu.br (A.M.P.); 4 LAPI-Laboratory of Research in Infectology, University Hospital Professor Edgard Santos (HUPES), Federal University of Bahia (UFBA), Salvador 40110-060, BA, Brazil; crbrites@gmail.com (C.B.); saranunes02@hotmail.com (S.V.N.); 5 BIOINOVAR-Biotechnology Laboratories, Biocatalysis, Bioproducts and Bioenergy, Institute of Microbiology Paulo de Góes, Federal University of Rio de Janeiro (UFRJ), Rio de Janeiro 21941-902, RJ, Brazil; mansoldo@micro.ufrj.br (F.R.P.M.); abvermelho@micro.ufrj.br (A.B.V.); 6 Department of Genetics, Luiz de Queiroz College of Agriculture, University of São Paulo (USP/ESALQ), Piracicaba 13418-900, SP, Brazil; thais.cataldi@usp.br

**Keywords:** COVID-19 screening, metabolomics, saliva, low-severity, mass spectrometry, machine learning

## Abstract

Omics approaches were extensively applied during the coronavirus disease 2019 (COVID-19) pandemic to understand the disease, identify biomarkers with diagnostic and prognostic value, and discover new molecular targets for medications. COVID-19 continues to challenge the healthcare system as the virus mutates, becoming more transmissible or adept at evading the immune system, causing resurgent epidemic waves over the last few years. In this study, we used saliva from volunteers who were negative and positive for COVID-19 when Omicron and its variants became dominant. We applied a direct solid-phase extraction approach followed by non-target metabolomics analysis to identify potential salivary signatures of hospital-recruited volunteers to establish a model for COVID-19 screening. Our model, which aimed to differentiate COVID-19-positive individuals from controls in a hospital setting, was based on 39 compounds and achieved high sensitivity (85%/100%), specificity (82%/84%), and accuracy (84%/92%) in training and validation sets, respectively. The salivary diagnostic signatures were mainly composed of amino acids and lipids and were related to a heightened innate immune antiviral response and an attenuated inflammatory profile. The higher abundance of thyrotropin-releasing hormone in the COVID-19 positive group highlighted the endocrine imbalance in low-severity disease, as first reported here, underscoring the need for further studies in this area.

## 1. Introduction

During the coronavirus disease 2019 (COVID-19) pandemic, omics approaches played a crucial role in elucidating the disease’s mechanisms, identifying biomarkers for diagnosis and prognosis, and discovering potential therapeutic targets [1,2,3]. In an endemic scenario, biomarkers for screening, monitoring, and early diagnosis of COVID-19 remain crucial for immunosurveillance.

The COVID-19 disease continues to challenge the healthcare system as the virus mutates, becoming more transmissible or better at evading the immune system. Since Omicron and its subvariants became dominant at the end of 2021, new and breakthrough cases have caused multiple infection waves in several countries [4,5]. In Brazil, the third wave of infections was driven by Omicron variants, initially increasing COVID-19 cases and deaths [6]. Most infections lead to asymptomatic or mild to moderate illness [7]. However, even this infection profile has been associated with critical health issues, such as long-COVID [8], which is characterized by a combination of clinical sequelae including pulmonary, neurological, dermatological, cardiac, renal, endocrine, and inflammatory conditions. These symptoms can last weeks, months, or even years after the initial infection [9].

Mild COVID-19 cases have also been linked to lingering symptoms and elevated need for primary care [8,10]. However, in general, Omicron infection was associated with lower hospital admission rates than previous severe acute respiratory syndrome coronavirus 2 (SARS-CoV-2) lineages [11]. Post-Omicron COVID-19 hospitalizations were related to primary pulmonary manifestation, other clinical manifestations, and incidental COVID-19 cases (i.e., where COVID-19 is not the primary reason for admission) [12,13,14,15]. Incidental cases were associated with shorter hospital stays, although still causing significant morbidity and substantial use of hospital resources [16]. Following the Omicron surge, hospital-acquired COVID-19 infections increased [17,18], raising the risk for vulnerable groups [19], such as elderly patients and those with other health issues, as they are more likely to need intensive care and have worse outcomes [12].

Even in the context of fewer virulent variants and increasing population immunity, Omicron infections were more common and severe than influenza and respiratory syncytial viruses in emergency departments [20,21]. Previous studies indicate that COVID-19 may also increase susceptibility to viral, bacterial, and fungal respiratory co-infections, which could complicate patient care, leading to hospitalization and extensive antimicrobial therapy [22,23,24].

Thus, COVID-19 screening in hospital settings allows for quick isolation, prevents transmission, and enables the timely initiation of antiviral therapies for those at risk of severe disease, as well as the protection of other vulnerable, non-infected patients. This information is crucial for appropriate hospital resource allocation and service planning for potential outbreaks.

Although reverse transcription-quantitative polymerase chain reaction (RT-qPCR) remains the gold standard for diagnosing SARS-CoV-2 infection, numerous tests using various analytical technologies to explore different biological matrices emerged after the pandemic outbreak, in response to the resource scarcity faced during the initial waves of COVID-19 [25,26]. In this context, saliva emerged as an efficient biological sample for detecting SARS-CoV-2 via molecular tests, achieving similar or superior performance to swab-based sampling methods [27,28,29,30]. Since then, several protocols and saliva-based tests have been approved or authorized for emergency use by regulatory agencies such as the Food and Drug Administration [31,32,33].

Previous studies found that saliva is not only a primary carrier of SARS-CoV-2 virus [34,35] but also undergoes substantial molecular changes in response to COVID-19 infection [36]. These changes are essential for understanding the pathophysiology of the disease and identifying potential biomarkers [37,38,39,40].

Saliva is also an easy-to-obtain and self-collectible sample, offering an additional advantage over invasive methods, such as nasopharyngeal/oropharyngeal swabs and blood collection, which are less acceptable to volunteers. Although saliva composition is highly variable and can be affected by external factors, it has been successfully applied for the diagnosis and physiological monitoring of diseases [41,42]. In addition to chronic diseases, such as oral cancer, diabetes, obesity, and Alzheimer’s disease [43,44,45,46], salivary markers have demonstrated good performance in discriminating viral infectious diseases, such as influenza [47], tuberculosis [48], human immunodeficiency virus [49,50], and Zika virus [51].

Metabolomics is a powerful approach to biomarker discovery that can evaluate complex phenotypes in response to physiological changes. Advances in mass spectrometry (MS)-based metabolomic strategies have led to notable progress in the discovery of metabolite-based biomarkers [52]. This approach has been established as a hypothesis-generating technique because of its considerable sensitivity and ability to detect thousands of metabolites simultaneously. Metabolome-wide changes in host metabolism have been linked to various aspects of COVID-19 pathophysiology and disease progression [1,39,53,54,55,56]. While some metabolic changes remained consistent across different waves of the COVID-19 pandemic, metabolic dysregulation induced by SARS-CoV-2 infection is influenced by different SARS-CoV-2 sublineages, clinical presentations, and therapeutic approaches [57]. This underscores the need to understand how past and emerging infection profiles affect the host metabolism. However, only a limited number of MS-based metabolomic studies have investigated the metabolic profile of saliva in the context of COVID-19, and most of these studies have included a small cohort of volunteers, particularly those with milder symptoms.

Therefore, we employed a data-driven approach to analyzing salivary metabolites from hospital-recruited volunteers, aiming to identify potential COVID-19-related signatures post-Omicron emergence. We performed direct solid-phase extraction, followed by non-target metabolomics, to explore a broad spectrum of metabolites and select those with potential for COVID-19 screening in hospital settings.

## 2. Results

### 2.1. Clinical Characteristics

Table 1 displays the clinical and pathological data of the study population recruited. Of the 174 participants, 100 were COVID-19-positive and 74 were COVID-19-negative. Gender distributions for the COVID-19-negative (male/female = 0.72) and -positive (male/female = 0.92) cohorts were similar. The average age differences were not statistically significant (40.0 ± 11.60 years for the COVID-19-negative group and 43.7 ± 14.88 years for the COVID-19-positive group; *p* = 0.175).

The average days between the onset of symptoms and saliva collection was 4.16 ± 1.80 for the COVID-19-negative group and 5.08 ± 2.56 for the COVID-19-positive group. At the time of saliva collection, 86% of the volunteers who tested positive for COVID-19 and 93% of those who tested negative were symptomatic. Most symptoms, except for fever (*p* < 0.03) and ageusia (*p* < 0.003), did not significantly differ between the two groups. In the COVID-19-positive group, 89% (*n* = 89) had mild disease and 11% (*n* = 11) had moderate disease.

Previous reports evidenced that pre-existing diseases are associated with disease evolution and poor clinical outcomes in volunteers with COVID-19 [58,59]. Due to recruitment, which included outpatients, some comorbidities were noticed among volunteers, such as diabetes, heart disease, and obesity. However, the proportion of patients with each comorbidity in each group was relatively low (less than 10%), except by immunosuppression, which was more frequent in the COVID-19-positive group (*n* = 24, *p* < 0.02).

### 2.2. Metabolomics Analysis

A set of 402 and 578 features was detected after data processing in the negative and positive ionization mode datasets, respectively. The clustering of quality control (QC) samples in the principal component analysis (PCA) score plot demonstrated the analytical quality of the analysis (Figure S1).

There was no significant difference in salivary features between the mild and moderate COVID-19 groups. Therefore, feature selection and classification models were developed based only on the presence or absence of the disease, as determined by the SARS-CoV-2 RT-qPCR test results (COVID-19-positive and COVID-19-negative groups).

The area under the curve (AUC) values of the top 100 selected features used to compose the models ranged from 0.58 to 0.79 for negative ion mode and 0.61 to 0.79 for positive ion mode. These two datasets of unidentified features generated eight different classification models built using random forest (RF), support vector machine (SVM), partial least squares-discriminant analysis (PLS-DA), and logistic regression (LR). The RF exhibited the best performance for both ionization modes (Tables S1 and S2). In the receiver operating characteristic (ROC) curve analysis of the RF models, the AUCs of the negative ion mode (Model I) and positive ion mode (Model II) were 0.936 (95% confidence interval [CI]: 0.86–0.98) and 0.941 (95% CI: 0.85–0.99), respectively (Figure S2A,B). The classification performances of Models I and II using the RF algorithm are listed in Table S3.

After feature identification, Models III and IV were built based only on the annotated compounds from Models I and II. Model III included 39 compounds from the negative ion mode data (Table S4), and Model IV comprised 24 compounds from the positive ion mode data (Table S5).

**Figure 1:**
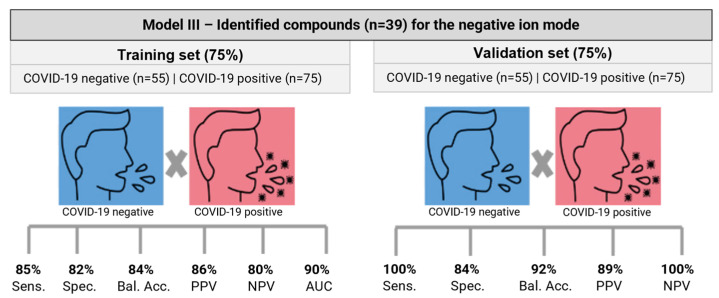
Model III performance metrics. Balanced accuracy (Bal. Acc.), sensitivity (Sens.), specificity (Spec.), negative predictive value (NPV), positive predictive value (PPV), and area under the ROC curve (AUC).

**Figure 2:**
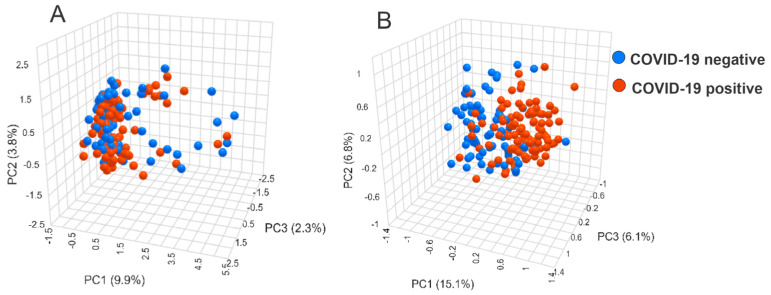
Three-dimensional principal component analysis (3D-PCA) score plots for salivary metabolites in COVID-19. (**A**) 3D-PCA scores plot of features (*n* = 402) detected in the negative ion mode. (**B**) 3D-PCA score plot of 39 metabolites from Model III after feature selection and compound annotation. The red dots represent the COVID-19-positive samples, and the blue dots represent the COVID-19-negative samples. PC: principal component.

Model III, from the negative ion mode, achieved the best performance, and its metrics are presented in Figure 1 and Figure S3. The less satisfactory results and metrics for Model IV are shown in Figure S5 and Table S6. PCA score plots for model III based on salivary metabolites before and after feature selection are presented in Figure 2. The algorithm improved the separation between COVID-19-positive and -negative groups.

In Model III, 11 out of 75 COVID-19-positive samples in the training set were erroneously classified as negative (Table S6). None of the 25 samples in the validation set were misclassified, resulting in high sensitivity (85% and 100%) and negative predictive values (80% and 100%) for the training and validation sets, respectively. Among the negative samples, 10 of 55 were misclassified in the training set, but only 3 out of 19 were misclassified as positive in the validation set (Table S6). This performance translated into positive predictive values (PPVs) of 86% for the training set and 89% for the validation set, with specificities of 82% and 84%, respectively. The metabolite panel correctly classified 89 out of 100 volunteers (89%) as positive for COVID-19 and correctly classified 61 out of 74 (82%) volunteers as COVID-19-negative.

Note that, in the training set, nine volunteers classified as “false positives” had two to six mild symptoms, and only one was asymptomatic. All volunteers classified as “false negatives” had at least one to four symptoms. In the validation set, volunteers considered “false positives” (*n* = 3) presented one or two symptoms during sample collection.

As comorbidities can act as both a causative and confounding factor, we also investigated whether there was any correlation between the metabolites in Model III and the comorbidities of patients in the COVID-19 positive and -negative cohorts.

As a result, no comorbidity showed a significant correlation with any predictor in the selected model, with all *p*-values corrected by the Bonferroni method presenting a value greater than 0.05. This may be influenced by the relatively low prevalence of each comorbidity within the groups. Notably, besides immunosuppression, which was more prevalent in the COVID-19-positive group (*n* = 24, *p* < 0.022), comorbidities did not differ significantly between the groups.

**Figure 3:**
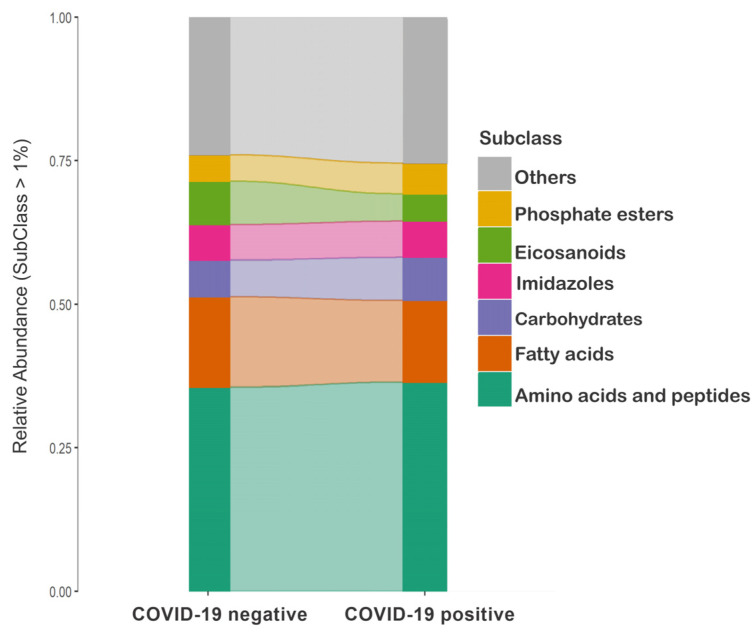
Alluvial plot depicting the relative abundance of the leading chemical subclasses from Model III in the COVID-19-negative and -positive groups.

The metabolites comprising Model III were mainly identified as amino acids, peptides, fatty acids, carbohydrates, imidazoles, eicosanoids, and phosphate esters (Figure 3 and Table S4). The alluvial plot and PCA biplot (Figure S4) demonstrate group variations. The vectors in Figure S4 indicate the directions in which the metabolites tended to vary, showing that a subset of the metabolites was drawn toward each group.

Table S4 lists all identified metabolites, highlighting 37 compounds that were significantly altered according to the Wilcoxon rank-sum test, with a false discovery rate (FDR) ≤0.05. Among the amino acids and peptides, L-histidine, L-arginine, L-glutamic acid, hydroxypropyl-proline, thyrotropin-releasing hormone (TRH), 1-methylhistidine, O-succinyl-L-homoserine, and N-phenylacetylaspartic acid stand out. The key lipid molecules include 2-hydroxyundecanoate, pentadecenoic acid, palmitoleic acid, 4-hydroxyoctanedioylcarnitine, azelaic acid, 2-amino-8-oxo-9,10-epoxy-decanoic acid, hydroxyoctanoic acid, dodecadienoic acid, LPC 6:0, 20-trihydroxy-leukotriene-B4 (20-OH(,3)-LTB4), PGF2alpha-11-acetate, and C17 sphinganine-1-phosphate (C17 S1P).

## 3. Discussion

Our study highlights the potential use of salivary metabolites to guide COVID-19 screening in hospital settings. The RF model, composed of 39 metabolites, enabled the differentiation of infected patients with sensitivity, specificity, and balanced accuracy of 100%, 84%, and 92% in the validation set, respectively. These results were obtained using a SARS-CoV-2 infection-negative control group, with 93% of the volunteers presenting COVID-19-like symptoms. Our study stands out for its superior performance in distinguishing symptomatic hospital-recruited controls from patients with COVID-19. Differentiating between these groups is particularly challenging, owing to their similar phenotypes. This surpasses previous studies [40,57], in which salivary metabolites had lower performance (i.e., sensitivities: 74–78% and specificities: 75–83%).

Metabolomics and machine learning algorithms have recently advanced, providing new approaches for identifying molecular markers to diagnose viral infections. These techniques were extensively explored during the COVID-19 pandemic. For instance, salivary metabolites were effective in distinguishing between severe and low-risk COVID-19 cases [37] and differentiating hospitalized patients from outpatients, helping to prevent unnecessary hospitalizations [38]. Moreover, a salivary metabolite, kynurenine, was identified as a potential marker for diagnosing and monitoring both long- and post-COVID syndrome [60]. However, in general, salivary metabolites were shown to be more severity-specific than disease-specific but performed well in models with healthy controls [39].

Compared to previous analyses, our study applied a larger mild COVID-19 cohort, mainly because of the collection period (from January 2022 to July 2022), during which Omicron and its variants dominated the epidemiological scenario. Indeed, mild illness represents more than 80% of cases [61], being the largest clinical manifestation of new and breakthrough infections.

As previously hypothesized by Frampas et al. [37] and evidenced in our data, mild COVID-19 was associated with limited and discrete alterations in the salivary metabolome compared to symptomatic controls. This limitation was even more pronounced when mild and moderate clinical presentations were compared, with no statistically significant differences between them. However, our positive cohort comprised a much smaller number of volunteers with moderate disease (*n* = 11) than those with mild disease (*n* = 89) at the time of sample collection.

In contrast to earlier findings, we demonstrated that three of the four best models achieved excellent performances in distinguishing low-severity COVID-19 from hospital-recruited controls. For translational purposes, we explored a model composed of only annotated metabolites (Model III). Although feature reduction caused by the identification bottleneck [62] affected the model’s performance, the panel includes metabolites that showed potential clinical applicability, highlighting an important biosignature for low-severity COVID-19.

We identified at least six affected metabolite subclasses that predominantly contributed to COVID-19 classification and explained approximately 75% of the abundance variation within groups. These compounds include amino acids, peptides, fatty acids, eicosanoids, and phosphate esters.

Previous reports indicate that patients with COVID-19 exhibit disrupted levels of various amino acids, including those involved in protein degradation and synthesis [55]. These disruptions were more pronounced in the critically ill and vulnerable patients. Specifically, low glutamine and isoleucine levels are associated with a higher mortality risk, whereas phenylalanine and cysteine levels can predict adverse outcomes [54].

In our data, histidine achieved the highest AUC and one of the highest fold changes among the annotated metabolites. Histidine is an essential amino acid involved in various physiological and immunological processes, including promoting antibody production in lymphocytes and the degranulation of mast cells and neutrophils [63]. It also serves as a precursor to histamine, a classical pro-inflammatory mediator [64]. Consistent with our findings, higher plasma histidine and 1-methylhistidine levels have been observed in volunteers infected with the Omicron variant. These levels are linked to the modulation of the immune response to this SARS-CoV-2 sublineage infection, contributing to the manifestation of mild symptoms [65]. This effect can be attributed to histidine’s ability to mitigate oxidative stress and reduce the production of TNF-α in neutrophils [66,67].

Arginine, a precursor of nitric oxide (NO), modulates signal transduction pathways in immune cells and regulates T-cell metabolism [68]. This alpha amino acid is involved in nitrogen synthesis via the urea cycle and creatine and polyamine synthesis [69], which are crucial for the host immune response and are recruited for the viral replication cycle [70]. Arginine deficiency substantially compromises infection resistance by disrupting NO synthesis [71]. Salivary arginine levels were lower in patients with severe COVID-19 and highly abundant in patients with mild COVID-19 [37,72], corroborating our findings. SARS-CoV-2 infection alters glutamine and glutamate metabolism [73], which are essential for synthesizing macromolecules, including glutathione, an essential antioxidant for preventing oxidative stress [74]. Elevated glutamate levels, indicating an increased use of glutamine and a low glutamine/glutamate ratio, are associated with a higher risk of COVID-19 infection and moderate/severe COVID-19 across various populations [73]. This metabolic impairment can persist for months after the infection [57]. In our metabolite panel, we identified lower levels of glutamate in the saliva of COVID-19-positive volunteers, suggesting a potential protective effect in low-severity cases.

In addition to amino acids, peptides, including free dipeptides and TRH, were highly represented in Model III. Most dipeptides were abundant in the COVID-19-positive group. This may be linked to a substantial impairment of salivary endopeptidases and their inhibitory activity during the disease, as reported in a proteomic study [75].

Endocrine dysfunction is also associated with SARS-CoV-2 infection [76]. Severe illness profoundly affects the hypothalamus–pituitary–thyroid (HPT) axis [77]. Notably, patients with severe COVID-19 exhibit higher levels of thyroid-related hormone abnormalities [78]. Cytokine signaling in the brain may directly suppress TRH and represents a critical event during the inflammatory process [79]. Mild-to-moderate COVID-19 is also associated with long-term thyroid dysfunction [80]. To our knowledge, this study is the first to report higher levels of tripeptide thyrotropin-releasing hormone in the saliva of volunteers with mild illnesses, suggesting an opposite effect in this group. This increase could be explained by the function of TRH extending beyond its role in regulating the HPT axis. It can also modulate immune cells, such as natural killer cells and T lymphocytes, acting in the innate and adaptive immune systems [81]. Therefore, further studies are required to understand the role of TRH in the progression of mild-to-moderate COVID-19.

Fatty acids constituted the second most abundant class of metabolites in the salivary signature, primarily medium- and long-chain fatty acids, including 2-hydroxyundecanoate, pentadecenoic acid, palmitoleic acid, 4-hydroxyoctanedioylcarnitine, hydroxyoctanoic acid, and dodecadienoic acid with significant changes in abundance in the positive cohort. Previous research has linked medium hydroxy fatty acids and 2-hydroxy fatty acids to anti-inflammatory and antiviral properties, including inhibiting viral replication [82,83,84] and reduction of inflammatory response via NFκB signaling inhibition in activated macrophages [85]. Higher levels of unsaturated fatty acids during coronavirus infection have also been associated with the inhibition of viral binding [56].

However, to the best of our knowledge, except for palmitoleic acid, whose levels have been correlated with a worse COVID-19 prognosis [56], no report has linked the previously mentioned fatty acids to the disease. This finding provides new insights into the host’s salivary response and points to additional investigations to understand its diagnostic and prognostic value for COVID-19.

Our panel also highlighted two eicosanoids as potential classifiers for low-severity COVID-19: PGF2alpha-11-acetate and 20-trihydroxy-leukotriene-B4 (20-OH(,3)-LTB4). Leukotrienes and prostaglandins are critical pro-inflammatory mediators in infectious diseases [86]. 20-OH(,3)-LTB4 is a product of omega oxidation of LTB4, a primary metabolite of polymorphonuclear leukocytes. The leukotriene pathway is crucial for the immune response to SARS-CoV-2 infection [87,88], associated with pathogenesis, disease severity, lung injury, and kidney damage [89]. High levels of leukotrienes, including LTB4, have been found in the serum and airway samples of patients with severe COVID-19 [87,88].

Additionally, the pro-inflammatory reprogramming of eicosanoids may contribute to long-term changes in the innate immune cell function in individuals with mild COVID-19 [90]. In our study, 20-OH(,3)-LTB4 was found to be lower in the COVID-19-positive group. Although the omega-oxidation products of leukotrienes are biologically less active [91], they display functions and binding properties similar to those of LTB4 on leukocytes [92]. Elevated basal levels of prostaglandins and other pro-resolving lipid mediators can also inhibit the innate and adaptive immune response activation [93]. Low levels of prostaglandins and leukotrienes may also indicate an attenuated inflammatory response, indicating low disease severity in the COVID-19-positive group.

Another notable lipid mediator in Model III was C17 sphinganine-1-phosphate, which was abundant in the COVID-19-positive group. Although not previously associated with COVID-19, sphinganine-1-phosphate showed anti-inflammatory properties by attenuating neutrophil infiltration in the kidneys and liver and reducing plasma levels of IL-6 and TNF-α [94]. Sphingolipid metabolites play a role against several viral diseases, including COVID-19 [95]. For example, sphingosine-1-phosphate (S1P) can prevent the SARS-CoV-2 spike protein from binding to its cellular receptor ACE 2, suggesting a potential role for sphingosine in inhibiting viral entry [96]. Ceramide-1-phosphate (C1-P) has also shown antiretroviral and immune-boosting properties, indicating its potential to control viral replication and resolve moderate-to-severe COVID-19 infections [97,98]. Salivary and plasma sphingosine levels were also inversely related with the severity of COVID-19, corroborating our findings [39].

In this study, we present a robust model for differentiating between mild-to-moderate COVID-19 volunteers and negative controls in a hospital setting, using “donor-friendly” saliva samples. We identified a metabolic signature that corroborated previously published plasma and salivary metabolomic phenotypes [1]. Additionally, a similar pattern was observed in the plasma metabolome following Omicron infection [65], suggesting that the salivary signature can reflect the systemic response of the host.

The main clinical usefulness of our study is that it provides a simple, sensitive, and minimally invasive tool for COVID-19 screening in hospital settings. It can allow for the quick isolation of infected patients and prevention of disease transmission, as well as timely initiation of antiviral therapies for those at risk of severe disease. However, as expected, the proportion of false positives was greater than that of false negatives, partially because of the recruitment of symptomatic volunteers to the negative cohort. This trend aligns with the reduced sensitivity of RT-qPCR in diagnosing low-severity illness [99].

Some limitations of the clinical findings of this study should be discussed. Hospital recruitment might introduce confounding factors, such as comorbidities and the use of medications, into the analysis. However, our analysis did not reveal any significant correlation between the metabolites from Model III and the comorbidities of the recruited volunteers. Further analysis in larger sample groups representing these comorbidities should be performed to better assess the potential impact of these factors. Also, symptomatic volunteers in the negative control group were not tested for other viral infections. Most clinical manifestations during the recruitment period were mild, and we did not adequately represent severe disease for inclusion in the screening model.

Despite maintaining a proportional distribution of sex and age averages between both groups, our cohort lacked adequate representation of elderly volunteers, who are more vulnerable to disease complications. However, we did not follow up on the clinical outcomes; therefore, the salivary biosignature only represents the moment it was collected. Salivary metabolites are strongly influenced by food consumption. Owing to hospital recruitment and ethical reasons, abstinence from food and drink for more than 30 min, as is generally recommended in studies with saliva, was not performed. This has been a common limitation during the COVID-19 pandemic [37,40]. Additionally, saliva is not the gold standard sample for the molecular diagnosis of COVID-19. However, it is a reliable alternative that is authorized and recommended by regulatory agencies, and various reports support its use in COVID-19 diagnosis [31,32,33].

As the volunteers were recruited between January and July 2022, we encourage comparisons with the new COVID-19 sublineages that have emerged since then to assess potential variations in salivary metabolite profiles. Finally, further validation and longitudinal studies in cohorts representing other respiratory infections, age groups, and disease severities should enhance the robustness and applicability of the panel.

## 4. Materials and Methods

### 4.1. Materials

HPLC-grade methanol (MeOH, LiChrosolv^®^) were purchased from Merck (Darmstadt, Germany), HPLC-grade acetonitrile (ACN) and formic acid were from Sigma-Aldrich (St. Louis, MO, USA). Ultrapure water (Milli-Q) was produced by the Milli-Q^®^ (Millipore, Bedford, MA, USA) purification system. Oasis PRiME HLB^®^ cartridges (3 mL; 60 mg) for solid-phase extraction were purchased from Waters Corporation, Milford, CT, USA.

### 4.2. Experimental Design and Study Population

Self-collected saliva samples were obtained from 174 volunteers between January and July 2022 at the Professor Edgard Santos Hospital Complex (C-HUPES) at the Federal University of Bahia, Salvador, Brazil. This study was conducted according to the principles of the Declaration of Helsinki and approved by the Research Ethics Committee of the Climério de Oliveira Maternity Unit at the Federal University of Bahia (protocol number 31748320.3.0000.5543 from 22 May 2020).

Samples were collected from outpatients from different C-HUPES ambulatory care facilities and healthcare professionals with either clinical suspicion of COVID-19 infection or known exposure to someone with COVID-19. The maximum interval between symptom onset and saliva collection was 15 days. The diagnosis of COVID-19 was confirmed by RT-qPCR in all patients. Viral RNA was extracted from the collected saliva using the QI-Aamp^®^ RNA Mini Kit (QIAGEN, Hilden, Germany). Amplification followed the Charité–Berlin protocol [100], validated for saliva samples [101], using an Applied Biosystems 7500 Real-Time PCR System. A positive result was defined as threshold cycle values of ≤40 for all target genes (E and RdRP).

Participants were categorized into COVID-19-positive and -negative groups based on their SARS-CoV-2 RT-qPCR results. Disease severity was classified based on the National Institutes of Health COVID-19 guidelines [102]. Demographic and clinical information were collected during saliva sampling.

### 4.3. Sample Collection and Processing

Saliva was self-collected following the previously described protocol [101]. Volunteers were instructed to refrain from eating, drinking, or using cream or mouthwash for 30 min before sampling. Participants were instructed to spit approximately 2 mL of saliva into sterile 30 mL urine cups. Samples were then homogenized, diluted with 1× phosphate-buffered saline (1:1, *v*/*v*) for the SARS-CoV-2 RT-qPCR test, and stored at −80 °C. Saliva samples were heat-inactivated after collection (65 °C, 30 min) [103] in an oven placed inside a Class II Biological Safety Cabinet. Subsequently, the samples were aliquoted (300 μL) and centrifuged (10,000× *g* at 4 °C for 10 min). The supernatants were recovered and frozen for further extraction.

### 4.4. Salivary Metabolites Extraction

Solid-phase extraction using Oasis PRiME HLB^®^ cartridges (3 mL; 60 mg) (Waters) was employed for saliva sample preparation, with a previously described modified method [104]. Briefly, saliva (300 μL) was diluted in 700 μL of Milli-Q water (H_2_O) and applied to the cartridge. After loading the sample (1 mL), the washing step was performed with 500 μL of H_2_O/MeOH (95:5, *v*/*v*), followed by two extraction steps with 500 μL of ACN/MeOH (90:10, *v*/*v*). The combined eluates were lyophilized (Enterprise I, Terroni, Brazil) and reconstituted in 360 μL of H_2_O/MeOH (50:50, *v*/*v*). Each sample (20 μL) was collected to form a pooled sample for QC. A QC sample was initially used for LC-MS/MS system stabilization and was inserted every 10 samples to check for extraction and system stability deviations. All saliva samples were extracted and analyzed randomly to minimize technical and instrumental biases.

### 4.5. Metabolomics Analysis Using LC-MS/MS

For untargeted metabolomic analysis, we utilized an Acquity H-Class (Waters^®^, Manchester, UK) coupled with a XEVO-G2XS Quadrupole Time-of-Flight (QToF) mass spectrometer (Waters), equipped with an Electrospray Ionization (ESI) source. The mass spectrometer was operated in both positive and negative ion modes (MS [+] and MS [−]). Chromatographic separation was performed using an ACQUITY UPLC ^®^ BEH AMIDE column (2.1 mm × 100 mm × 1.7 μm, Waters). Mobile phase A consisted of ACN and 0.1% formic acid, whereas mobile phase B comprised Milli-Q water and 0.1% formic acid. The flow rate was set to 0.4 mL min^−1^. Initially, the column was conditioned with 5% B, ramped to 70% B for 10 min, and held for 1 min. Mobile phase B returned to 5% within 0.1 min and equilibrated for 3.9 min before the next injection, making the total run time 14 min. The injection volume was 2 μL for both MS [−] and MS [+] modes.

The instrument was operated in MS^E^ mode across the *m*/*z* range of 100–1000 Da, with an acquisition time of 0.5 s per scan. Operational parameters included a source temperature of 140 °C, desolvation temperature of 550 °C, desolvation gas flow of 900 L h^−1^, capillary voltages of 3.0 kV (+)/2.5 kV (−), and a cone voltage of 40 V. MS^E^ analysis utilized a collision energy of 6 V for low-energy scans and ramped from 20 to 50 V for high-energy scans. Leucine enkephalin (555.62 Da; 200 pg μL^−1^) served as a lock mass for accurate mass measurements, and a 0.5 mmol L^−1^ solution of sodium formate was used for mass calibration.

### 4.6. Data Processing and Putative Identification of Metabolites

LC-MS/MS raw files were imported into Progenesis™ QI software version 2.4 (Nonlinear Dynamics, Newcastle, UK) for data processing, including adduct selection, peak alignment, and deconvolution, as well as compound annotation based on MS^E^ experiments. For the data acquired in positive ion mode, the adducts considered were [M + H]^+^, [M + K]^+^, [M + Na]^+^, [M + H − 2H_2_O]^+^, and [M + H − H_2_O]^+^. In the negative ion mode, [M − H]^−^, [M + Cl]^−^, [M − H_2_O − H]^−^, and [M + FA − H]^−^ were used.

Due to the acquisition of either low or high energy, information on precursor ions (mass error of ≤ 5 ppm) and fragments (mass error of ≤ 10 ppm) coexisted within the same mass spectrum. The identification of metabolites relied on MS1 and MS2 experiments [105]. The evaluation criteria for validating the annotated molecules included fragmentation profiles, mass accuracies, mass errors, isotope similarities, and physiological roles. External SDF-based spectral libraries such as LipidMaps (http://www.lipidmaps.org/), Human Metabolome Database (http://www.hmdb.ca/metabolites), and the MassBank of North America (https://mona.fiehnlab.ucdavis.edu/) were used (all accessed on 12 July 2023). To enhance compatibility between Progenesis PQI data and these external SDF-based spectra libraries, increasing the number of fragment matches, we used an in-house, freely available, open-source software called “SDF2PQI” (https://github.com/pedrohgodoys/sdf_to_pqi, accessed on 15 August 2023) [106]. Metabolite identification criteria were based on previous studies by Sah et al. and Liebisch et al. [107,108].

### 4.7. Features Selection and Classification Models

**Figure 4:**
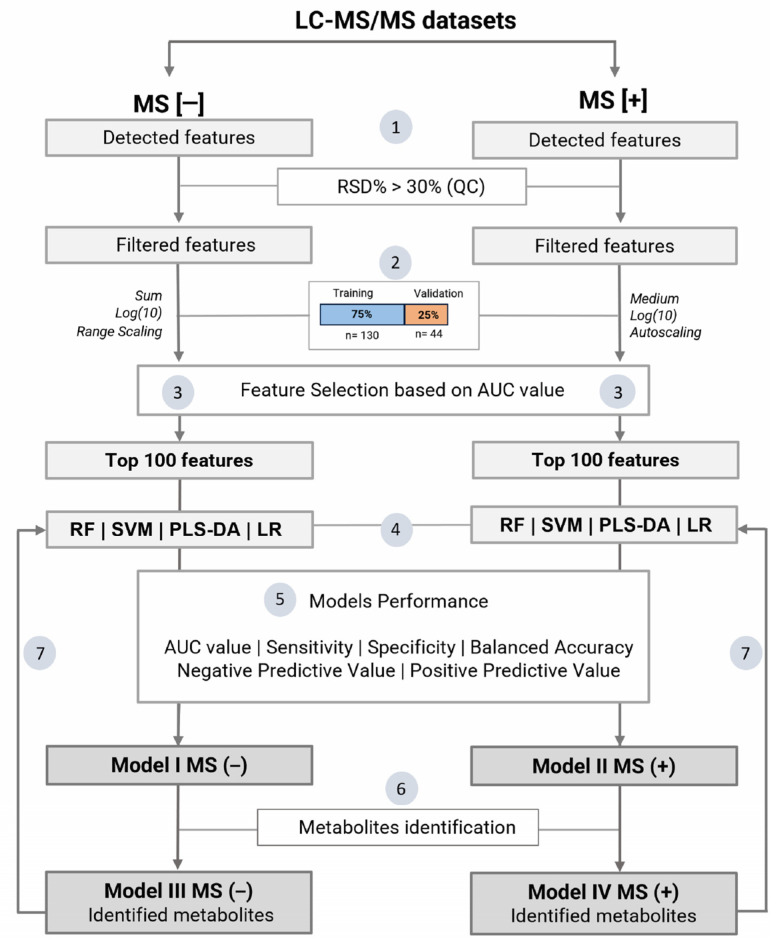
The data processing workflow. (1) Features detected in LC-MS/MS analysis from negative and positive ionization modes. (2) Only features with an RSD < 30% were retained in the final data matrix. The samples were randomly divided into a training set (*n* = 130, [75%]) and a validation set (*n* = 44, [25%]). (3) Feature selection was performed based on the individual AUC value. (4) For each ionization mode, four algorithms (PLS-DA, SVM, RF, and LR) were used to build classification models using the top 100 selected features. (5) The best models were selected based on the AUC value, sensitivity, specificity, balanced accuracy, and positive and negative predictive values, obtained using confusion matrix data. (6) Metabolites identification of the top 100 features. (7) New classification models were built only with identified metabolites. RSD: relative standard deviation; AUC: area under the curve; PLS-DA: partial least squares discriminant analysis; SVM: support vector machine; RF: random forest; LR: logistic regression; ROC curve: receiver operating characteristic curve.

To develop a model distinguishing between negative and positive COVID-19 groups, we utilized the web platform MetaboAnalyst™ 5.0 in the “Biomarker Analysis” module [109]. Figure 4 outlines the data processing workflow used in this study. Two datasets from the analytes acquired in MS [+] and MS [−] modes were used to build the models. Initially, the data were uploaded and filtered using the relative standard deviation (RSD) for intra-batch QC samples. Analytes with RSD > 30% were excluded from statistical modeling. Samples were randomly divided into a training set [*n* = 130 [75%]; COVID-19 positive (*n* = 75) and COVID-19 negative (*n* = 55)] and a validation set [*n* = 44 [25%]; COVID-19 positive (*n* = 25) and COVID-19 negative (*n* = 19)]. Random partitioning ensured that the sample proportions in both subsets for the two conditions analyzed were similar to the total set proportion (0.74).

For the positive ion mode, the data were median-normalized, log-transformed, and scaled by autoscaling, whereas, for the negative ion mode, the data were sum-normalized, log-transformed, and scaled by range scaling. To select features capable of classifying the presence of COVID-19, we employed individual AUC values and selected the top 100 features from each ionization mode to create the models. This strategy aims to limit the features used, eliminate noise, and reduce overfitting [110]. Each selected feature group was tested with four algorithms—RF, SVM, PLS-DA, LR—to evaluate their performance in discriminating between COVID-19-positive and -negative groups. Model performance metrics, including sensitivity, specificity, balanced accuracy, negative predictive value (NPV), and positive predictive value (PPV), were assessed using confusion matrix data. The best model for each ionization mode was selected for feature annotation. After compound identification, similar classification models were built specifically for the identified compounds (Models III and IV).

Identified metabolites were deemed significantly altered when *p*-values were <0.05 and FDR < 0.05 (Wilcoxon rank-sum test). For visualization purposes, PCA, PCA biplot, and fold-change analysis were performed to evaluate how the selected model contributed to generating the hypotheses for the conditions studied.

A metabology approach was employed to determine the abundance of chemical classes in both COVID-19-positive and -negative groups, involving metabolomic analysis using chemical ontology information from community ecology tools [111]. Briefly, previously annotated metabolites were categorized using ClassyFire for ontological classification, and the relative abundances of chemical classes from Model III were computed and visualized in an alluvial plot (Figure 3).

### 4.8. Statistical Analysis

The Shapiro–Wilk test was used to assess the normality of the data distribution. Student’s *t*-test or the Mann–Whitney *U* test was used to compare continuous variables between COVID-19-positive and -negative groups and to compare metabolite profiles between the mild and moderate COVID-19 groups (*p* < 0.05 and FDR < 0.05) before constructing classification models. The chi-squared test or Fisher’s exact test was used to compare categorical variables between COVID-19-positive and -negative groups.

The association of comorbidities with the features selected by the models was verified using the rcorr (Matrix of Correlations and *p*-values) function of the Hmisc package (version 5.1-3) [112], with the *p*-values corrected by the Bonferroni method using the *p*.adjust function from R stats in R (version 4.4.1; R Core Team, 2024).

## 5. Conclusions

Our data emphasize the potential use of saliva to identify COVID-19 biosignatures in mild-to-moderate disease. The final model, based on the RF algorithm, demonstrated outstanding performance in screening for low-severity COVID-19 in hospital settings. The salivary panel revealed a distinctive signature mainly comprising amino acids, peptides, fatty acids, and lipid mediators, highlighting an enhanced innate immune antiviral response and an attenuated inflammatory profile. The higher abundance of TRH in the positive COVID-19 group also indicates an endocrine imbalance in low-severity diseases, as reported here for the first time. Numerous significant changes in amino acid and lipid species, along with the identification of new COVID-19-related metabolites, provide new insights into the host salivary response and pathophysiological processes contributing to mild illness.

## Figures and Tables

**Table 1:** Basic demographics of the study population.

Variable	COVID-19 Positive	COVID-19 Negative	Total	*p*-Value *
Volunteers, n (%) Age (years) Male/Female, n (%)	100 (57.5) 43.71 ± 14.88 48 (48)/52 (52)	74 (42.5) 40.09 ± 11.60 31 (41.9)/43 (58.1)	174 (100) 42.18 ± 13.57 79 (45.4)/95 (54.6)	0.048 0.175 0.425
Comorbidities, n (%)				
Diabetes Heart disease Hypertension Obesity Kidney disease Immunosuppression Respiratory disease	9 (9) 8 (8) 0 1 (1) 5 (5) 24 (24) 3 (3)	7 (9.4) 3 (4) 1 (1.3) 5 (6.8) 0 7 (9.4) 1 (1.3)	16 (9.2) 11 (6.3) 1 (0.6) 6 (3.4) 5 (2.9) 32 (18.4) 4 (2.3)	0.920 0.357 0.425 0.080 0.070 0.022 0.637
The onset of symptoms to sample collection (days)	5.08 ± 2.56	4.16 ± 1.80	4.68 ± 2.30	0.045
Symptoms, n (%)				
Cough Sore throat Fever Coryza Headache Myalgia Dyspnea Nasal congestion Ageusia Fatigue or Weakness Diarrhea Sneezing Anosmia Nausea and vomiting Abdominal Pain Chills	62 (62) 38 (38) 37 (37) 30 (30) 33 (33) 14 (14) 11 (11) 9 (9) 9 (9) 7 (7) 4 (4) 3 (3) 2 (2) 2 (2) 2 (2) 2 (2)	36 (48.6) 25 (33.8) 11 (14.9) 45 (60.8) 32 (43.2) 13 (17.6) 7 (9.4) 10 (13.5) 20 (27) 11 (14.9) 3 (4) 2 (2.7) 5 (6.8) 2 (2.7) 1 (1.3) 3 (4)	98 (56.3) 63 (36.2) 48 (27.6) 75 (43.1) 65 (37.4) 27 (15.5) 18 (10.3) 19 (10.9) 29 (16.7) 18 (10.3) 7 (4) 5 (2.9) 7 (4) 4 (2.3) 3 (1.7) 5 (2.9)	0.109 0.679 0.003 0.090 0.221 0.666 0.937 0.485 0.003 0.152 0.989 1.000 0.136 1.000 0.575 1.000
Disease severity, n (%)				
Mild Moderate	89 (89) 11 (11)	- -	89 (89) 11 (11)	*-* *-*

Data were presented as mean ± standard deviation or number (percentage). * *p*-values were calculated using the Mann–Whitney *U* test for continuous variables and the chi-squared test (or Fisher’s exact test) for categorical variables.

## Data Availability

The metabolomics data have been deposited at the MetaboLights repository (accession number MTBLS10719).

## Supplementary Materials

The following supporting information can be downloaded from https://www.mdpi.com/article/10.3390/ijms252211899/s1.
