# Supplementary material for: Mass Spectrometry-Based Metabolomics Reveals a Salivary Signature for Low-Severity COVID-19
Source: Int J Mol Sci. 2024 Nov 6;25(22):11899. doi: 10.3390/ijms252211899 (PMC11593410; doi:10.3390/ijms252211899)
Supplement: Supplementary file 1 [file ijms-25-11899-s001.zip › ijms-3133505-supplementary.pdf]

Supporting Information for:

Mass Spectrometry-Based Metabolomics Reveals a Salivary Signature for Low-Severity COVID-19

*Iasmim Lopes de Lima[1,2†], Alex Ap. Rosini Silva[3,†], Carlos Brites[4], Natália Angelo da Silva Miyaguti[3], Felipe Raposo Passos Mansoldo[5], Sara Vaz Nunes[4], Pedro Henrique Godoy Sanches[3], Thais Regiani Cataldi[6], Caroline Pais de Carvalho[1,2], Adriano Reis da Silva[1,2] , Jonas Ribeiro da Rosa[3], Mariana Magalhães Borges[1,2], Wellisson Vilarindo Oliveira[1,2], Thiago Cruz Canevari[1], Alane Beatriz Vermelho[5], Marcos Nogueira Eberlin[1,2 *], Andreia de Melo Porcari[3]*

1 PPGEMN, School of Engineering, Mackenzie Presbyterian University, São Paulo, SP 01302-907, Brazil

2 MackGraphe - Mackenzie Institute for Research in Graphene and Nanotechnologies, Mackenzie Presbyterian Institute, São Paulo, SP 01302-907, Brazil

3 MS[4]Life Laboratory of Mass Spectrometry, Health Sciences Postgraduate Program, São Francisco University—USF, Bragança Paulista, SP 12916-900, Brazil

4 LAPI - Laboratory of Research in Infectology, University Hospital Professor Edgard Santos (HUPES), Federal University of Bahia (UFBA), Salvador, BA 40110-060, Brazil

5 BIOINOVAR − Biotechnology Laboratories: Biocatalysis, Bioproducts and Bioenergy, Institute of Microbiology Paulo de Góes, Federal University of Rio de Janeiro (UFRJ), Rio de Janeiro, RJ 21941-902, Brazil

6 Department of Genetics, Luiz de Queiroz College of Agriculture, University of São Paulo (USP/ESALQ), Piracicaba, SP 13418-900, Brazil

† These authors contributed equally to this work.

*Correspondence: Marcos Nogueira Eberlin (marcos.eberlin@mackenzie.br)

List of Contents

Figure S1. Principal Component Analysis (PCA) of Quality Control (QC), COVID-19 positive, and COVID-19 negative samples from data acquired in the negative (A) and positive (B) ion modes.

Table S1. Classification performance of machine learning algorithms on the negative ion mode data.

Table S2. Classification performance of machine learning algorithms on the positive ion mode data.

Figure S2. ROC curves for Models I and II, showing their average performance across all Monte Carlo cross-validation runs.

Table S3. Classification performance of Models I and II using Random Forest algorithm.

Table S4. Annotated metabolites from Model III.

Table S5. Annotated metabolites from Model IV.

Figure S3. ROC curve for Model III, showing its average performance across all Monte Carlo cross-validation runs.

Figure S4. PCA biplot analysis of 39 metabolites identified in Model III.

Figure S5. PCA score plots of the data detected in positive ionization mode for COVID-19-positive and -negative groups.

Table S6. Classification performance of Models III and IV using the Random Forest algorithm.

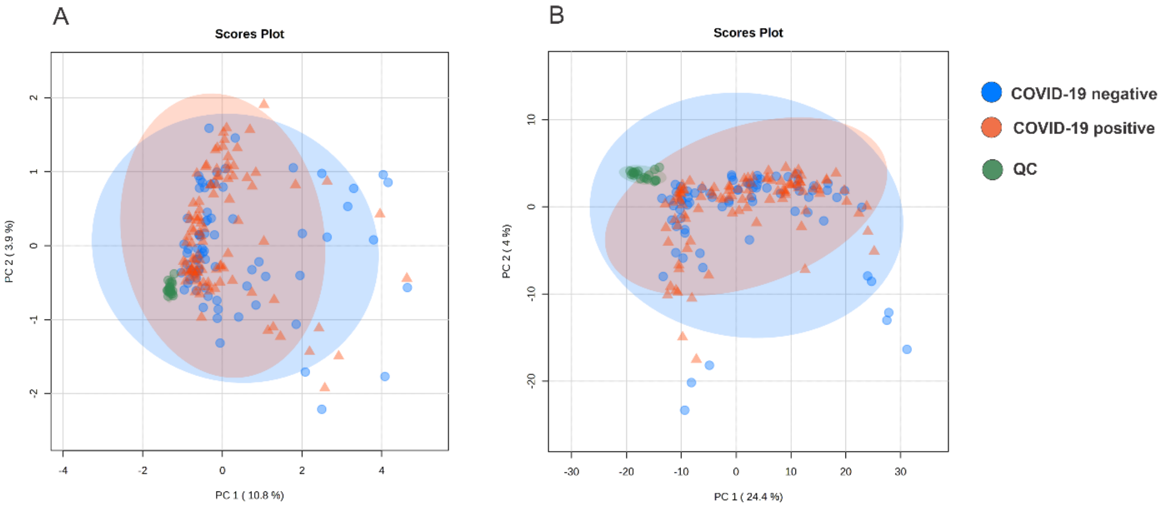

Figure S1. Principal Component Analysis (PCA) of Quality Control (QC), COVID-19 positive and COVID-19 negative samples from data acquired in the negative (A) and positive (B) ion modes. The orange triangles represent the COVID-19 positive samples, the blue dots represent the COVID-19 negative samples, and the green dots represent the QC samples. PC: Principal component.

Table S1. Classification performance of machine learning algorithms on the negative ion mode data

Negative Ion Mode - TOP 100 (AUC 0.58-0.79)								
Algorithm	**Random Forest**		**PLS-DA**		**SVM**		**Logistic regression**	
Dataset (130/44)*	Training	Validation	Training	Validation	Training	Validation	Training	Validation
True Negative	46	18	45	16	40	11	31	12
False Positive	9	1	10	3	15	8	24	7
False Negative	6	0	16	0	22	6	39	7
True Positive	69	25	59	25	53	19	36	18
Sensitivity	92%	100%	78%	100%	70%	76%	48%	72%
Specificity	84%	94%	81%	84%	72%	57%	56%	63%
Bal. accuracy	87%	97%	80%	92%	71%	66%	52%	67%
PPV	88%	96%	85%	89%	77%	70%	60%	72%
NPV	88%	100%	73%	100%	64%	64%	44%	63%

* The training set was composed of 130 volunteers (55 COVID-19 negative and 75 COVID-19 positive), while the validation set consisted of 44 volunteers (19 COVID-19 negative and 25 COVID-19 positive). AUC: Area Under the Curve, PLS-DA: Partial Least-Squares Discriminant Analysis, SVM: Support Vector Machine, PPV: Positive Predictive Value, NPV: Negative Predictive Value.

Table S2. Classification performance of machine learning algorithms on the positive ion mode data

Positive Ion Mode - TOP 100 (AUC 0.61-0.79)								
Algorithm	**Random Forest**		**PLS-DA**		**SVM**		**Logistic Regression**	
Dataset (130/44)*	Training	Validation	Training	Validation	Training	Validation	Training	Validation
True Negative	48	16	46	16	47	17	35	14
False Positive	7	3	9	3	8	2	20	5
False Negative	6	2	6	3	10	5	18	10
True Positive	69	23	69	22	65	20	57	15
Sensitivity	92%	92%	92%	88%	86%	80%	76%	60%
Specificity	87%	84%	83%	84%	85%	89%	63%	73%
Bal. accuracy	89%	88%	87%	86%	86%	84%	69%	66%
PPV	90%	88%	88%	88%	89%	90%	74%	75%
NPV	89%	89%	88%	84%	82%	77%	66%	58%

* The training set was composed of 130 volunteers (55 COVID-19 negative and 75 COVID-19 positive), while the validation set consisted of 44 volunteers (19 COVID-19 negative and 25 COVID-19 positive). AUC: Area Under the Curve, PLS-DA: Partial Least-Squares Discriminant Analysis, SVM: Support Vector Machine, PPV: Positive Predictive Value, NPV: Negative Predictive Value.

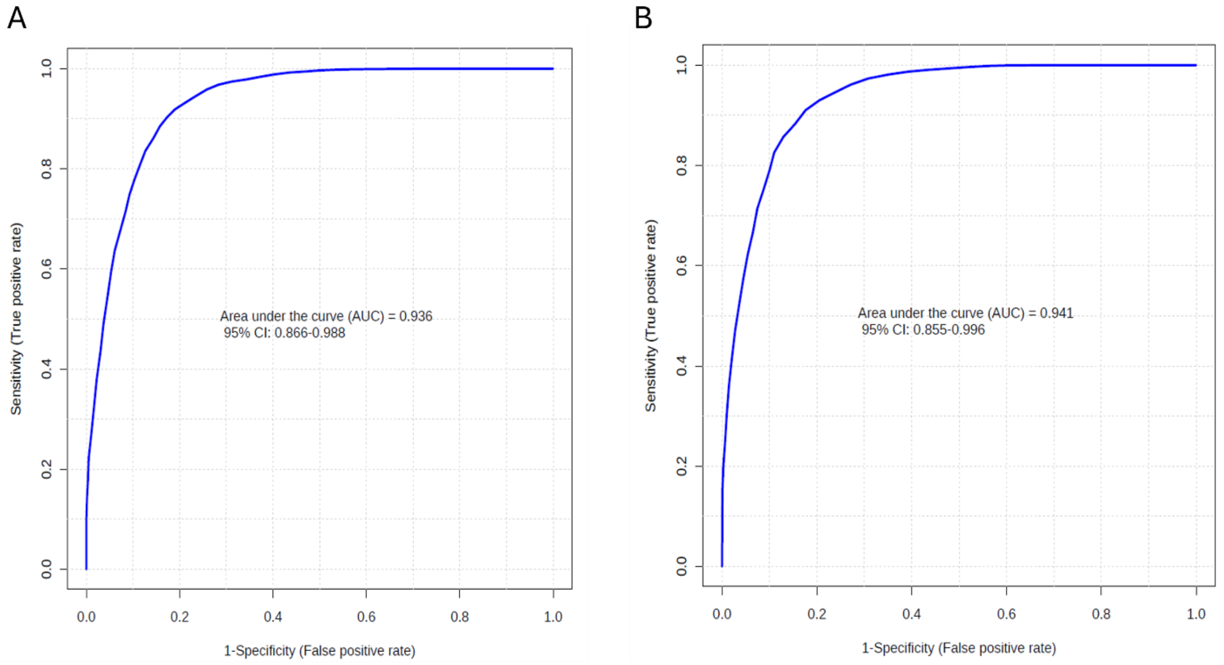

Figure S2. ROC curves for Models I and II, showing their average performance across all Monte Carlo cross-validation runs. A) ROC curve for Model I using the Random Forest algorithm. B) ROC curve for Model II using the Random Forest algorithm.

Table S3. Classification performance of Models I and II using the Random Forest algorithm

	**Model I - Negative Mode** 100 metabolites (AUC 0.58-0.79)		**Model II - Positive Mode** 100 metabolites (AUC 0.61-0.79)	
Algorithm	**Random Forest**		**Random Forest**	
Dataset (130/44)*	Training	Validation	Training	Validation
True Negative	46	18	48	16
False Positive	9	1	7	3
False Negative	6	0	6	2
True Positive	69	25	69	23
Sensitivity	92%	100%	92%	92%
Specificity	84%	94%	87%	84%
Bal. accuracy	87%	97%	89%	88%
VPP	88%	96%	90%	88%
VPN	88%	100%	89%	89%

* The training set was composed of 130 volunteers (55 COVID-19 negative and 75 COVID-19 positive), while the validation set consisted of 44 volunteers (19 COVID-19 negative and 25 COVID-19 positive). AUC: Area Under the Curve, PPV: Positive Predictive Value, NPV: Negative Predictive Value.

Table S4. Annotated metabolites from Model III

Metabolite n°	*m/z* measured	Rt (min)	Adducts	Formula	Description	Mass Error (*ppm*)	AUC Value	Fold Change	p-adjusted	Subclass
1	136.0512	0.53	M-H_2O-H	$C_6H_9N_3O_2$	L-Histidine	-2.80	0.79	1.44	1.03E-10	
2	209.0938	1.47	M-H_2O-H	$C_{10}H_{16}N_2O_4$	Hydroxyprolyl-Proline	2.69	0.79	1.21	5.40E-10	
3	361.1633	0.68	M-H	$C_{16}H_{22}N_6O_4$	Thyrotropin releasing hormone	0.84	0.75	1.57	9.73E-09	
4	209.0814	0.71	M+Cl	C_6H14N4O2	L-Arginine	1.60	0.72	1.20	5.85E-07	
5	228.0982	0.71	M-H	$C_9H_{15}N_3O_4$	Asparaginyl-Proline	-3.53	0.72	1.27	4.84E-06	
6	200.0565	2.50	M-H_2O-H	$C_8H_{13}NO_6$	O-Succinyl-L-homoserine	0.21	0.71	1.33	3.15E-06	
7	268.1297	2.01	M-H_2O-H	$C_{12}H_{21}N_3O_5$	N-Acetyl-Glycil-Valyl-Glycine Methylester	-1.96	0.69	1.36	1.41E-05	Amino acids and peptides
8	150.0666	1.27	M-H_2O-H	$C_7H_{11}N_3O_2$	1-Methylhistidine	-3.88	0.68	1.21	1.89E-04	
9	232.0625	2.50	M-H_2O-H	$C_{12}H_{13}NO_5$	N-Phenylacetylaspartic acid	4.05	0.68	1.19	1.68E-04	
10	252.0963	0.70	M+FA-H	$C_{11}H_{13}NO_3$	Acetyl-L-phenylalanine	0.25	0.66	1.12	2.05E-05	
11	128.0349	1.45	M-H_2O-H	$C_5H_9NO_4$	L-Glutamic acid	-3.00	0.65	0.53	8.89E-04	
12	415.2290	3.57	M-H	$C_{17}H_{32}N_6O_6$	Glutamyl-Leucyl-Arginine	-4.87	0.61	0.79	5.58E-02	
13	934.4565	6.74	M-H_2O-H	$C_{43}H_{71}N_9O_{11}S_2$	Cyclin D1	3.02	0.60	0.79	1.69E-01	
14	201.1488	0.71	M-H	$C_{11}H_{22}O_3$	2-Hydroxyundecanoate	-3.92	0.76	1.36	5.40E-10	
15	285.2061	0.71	M+FA-H	$C_{15}H_{28}O_2$	Pentadecenoic acid	-4.16	0.75	1.33	4.04E-08	
16	299.2218	0.70	M+FA-H	$C_{16}H_{30}O_2$	Palmitoleic acid	-3.86	0.74	1.13	1.45E-07	
17	259.1904	0.71	M-H	$C_{14}H_{28}O_4$	MG 11:0	-4.16	0.70	1.33	1.01E-07	
18	332.1702	4.08	M-H	$C_{15}H_{27}NO_7$	4-Hydroxyoctanedioylcarnitine	-3.76	0.70	1.03	9.71E-06	Fatty acids
19	187.0969	0.73	M-H	$C_9H_{16}O_4$	Azelaic acid	-3.42	0.68	1.16	8.09E-05	
20	260.0925	0.73	M+FA-H	$C_{10}H_{17}NO_4$	2-amino-8-oxo-9,10-epoxy-decanoic acid	-2.21	0.68	1.28	2.05E-05	
21	159.1020	0.71	M-H	$C_8H_{16}O_3$	Hydroxyoctanoic acid	-4.15	0.65	1.22	1.45E-03	
22	195.1384	0.63	M-H	$C_{12}H_{20}O_2$	Dodecadienoic acid	-3.55	0.60	0.48	1.26E-02	
23	248.9946	3.24	M+Cl	$C_5H_{11}O_7P$	5-Deoxyribose-1-phosphate	4.43	0.77	1.37	5.40E-10	Carbohydrates
24	202.0728	2.50	M-H_2O-H	$C_8H_{15}NO_6$	N-Acetyl-D-glucosamine	3.05	0.69	1.26	1.86E-05	
25	139.0506	2.50	M-H	$C_6H_8N_2O_2$	Imidazolepropionic acid	-4.75	0.70	1.07	3.90E-05	Imidazoles
26	121.0517	3.73	M-H_2O-H	$C_5H_8N_4O$	5-Aminoimidazole-4-carboxyamide	-2.01	0.66	1.18	1.07E-03	
27	395.2430	0.71	M-H	$C_{22}H_{36}O_6$	PGF2alpha-11-acetate	-2.26	0.64	0.70	1.98E-02	Eicosanoids
28	383.2058	4.41	M-H	$C_{20}H_{32}O_7$	20-Trihydroxy-leukotriene-B4	-4.38	0.63	0.73	3.15E-02	
29	412.2470	0.73	M+FA-H	$C_{17}H_{38}NO_5P$	C17 Sphinganine-1-phosphate	0.13	0.66	1.28	1.90E-04	Phosphate esters
30	203.1068	0.73	M-H_2O-H	$C_{13}H_{18}O_3$	Hexyl salicylate	-4.15	0.74	1.20	1.99E-07	Benzoic acids*
31	189.0766	1.02	M-H	$C_8H_{14}O_5$	Diethyl L-malate	-1.51	0.65	1.16	1.26E-02	Beta hydroxy acids*
32	225.1486	0.71	M-H_2O-H	$C_{13}H_{24}O_4$	1,4-Nonadiol diacetate	-4.34	0.66	1.06	3.90E-05	Fatty alcohol esters*
33	186.1128	0.73	M-H_2O-H	$C_9H_{19}NO_4$	Panthenol	-3.69	0.73	1.31	2.31E-07	Fatty amides*
34	400.1742	0.68	M+FA-H	$C_{14}H_{30}NO_7P$	LPC 6:0	0.06	0.65	1.09	5.25E-04	Glycerophosphocholines*
35	173.1175	0.71	M-H	$C_9H_{18}O_3$	FA 9:0;O	-4.85	0.78	1.05	5.73E-09	Medium-chain hydroxy acids*
36	237.1501	0.71	M-H_2O-H	$C_{14}H_{24}O_4$	Monomenthyl succinate	1.72	0.78	1.31	5.73E-09	Monoterpenoids*
37	149.0467	0.53	M-H	$C_6H_6N_4O$	7-Methylhypoxanthine	-1.54	0.73	1.27	2.40E-07	Purines*
38	251.9949	0.53	M+Cl	$C_6H_8N_3O_4P$	4-amino-2-methyl-5-phosphomethylpyrimidine	1.39	0.71	1.25	6.46E-06	Pyrimidines and pyrimidine*
39	257.0773	2.29	M-H	$C_{10}H_{14}N_2O_6$	5-Methyluridine	-2.38	0.66	0.68	5.28E-03	

Rt: Retention time;*others

Table S5. Annotated metabolites from Model IV

m/z measured	Rt (min)	Adducts	Formula	Description	Mass Error (*ppm*)	AUC Value	Fold Change	p-adjusted	Subclass
513.2561	6.61	M+H-2H$_2$O	$C_{24}H_{36}N_8O_7$	Thyrotropin-releasing hormone precursor	-1.43	0.64	1.42	1.67E-02	Amino acids and peptides
310.1391	4.14	M+H	$C_{14}H_{19}N_3O_5$	Tyrosyl-Glutamine	3.99	0.61	1.22	6.28E-02	
448.2969	5.73	M+H-H$_2$O	$C_{28}H_{39}N_3O_3$	N-Docosahexaenoyl Histidine	2.30	0.60	1.22	7.27E-02	
404.2776	6.47	M+Na	$C_{22}H_{39}NO_4$	N-Linoleoyl Threonine	1.29	0.59	1.37	1.34E-01	
407.2685	4.14	M+H-2H$_2$O	$C_{26}H_{38}N_2O_4$	N-Docosahexaenoyl Asparagine	-1.87	0.58	0.77	1.53E-01	
791.4476	4.64	M+Na	$C_{41}H_{69}O_{11}P$	PA 18:2/PGD2	-4.03	0.60	1.27	6.22E-02	Eicosanoids
938.6473	7.77	M+Na	$C_{50}H_{94}NO_{11}P$	PC 22:0/PGD1	1.82	0.55	1.77	3.11E-01	
977.5007	7.91	M+Na	$C_{49}H_{79}O_{16}P$	PI 20:4/PGE2	0.90	0.55	1.74	2.14E-01	
531.3065	2.88	M+Na	$C_{25}H_{49}O_8P$	PA 22:0	1.48	0.62	1.38	1.44E-02	Glycerophosphates
725.5505	7.66	M+H-H$_2$O	$C_{42}H_{79}O_8P$	PA 39:2	3.45	0.54	2.24	3.12E-01	
872.6721	6.89	M+H-H$_2$O	$C_{49}H_{96}NO_{10}P$	PS 43:0	-2.02	0.58	1.01	5.87E-01	Glycerophosphoserines
596.3941	6.15	M+H	$C_{29}H_{58}NO_9P$	LPS 23:0	3.20	0.55	1.20	2.87E-01	
342.19	6.61	M+H	$C_{17}H_{27}NO_6$	Deca-2,8-dienedioylcarnitine	-3.19	0.58	1.18	1.34E-01	Fatty acid
301.2544	5.41	M+H	$C_{18}H_{33}FO_2$	18-Fluorooleic acid	2.18	0.61	1.45	5.04E-02	
437.2367	0.77	M+H-2H$_2$O	$C_{24}H_{40}O_7S$	Ursodeoxycholate 3-sulfate	2.24	0.71	1.37	4.53E-05	Bile acids and derivatives
994.0864	7.77	M+K	$C_{31}H_{40}N_7O_{20}P_3S$	5-Hydroxyferuloyl CoA	-3.01	0.54	1.35	2.88E-01	Fatty acyl thioesters
237.2197	2.31	M+Na	$C_{14}H_{30}O$	Tetradecanol	3.99	0.75	1.18	6.11E-05	Fatty alcohols
652.4002	3.65	M+H-2H$_2$O	$C_{35}H_{62}NO_{10}P$	PE 30:4;O2	4.23	0.60	1.33	6.22E-02	Glycerophosphoethanolamines
647.3158	6.08	M+Na	$C_{29}H_{53}O_{12}P$	LPI 20:2	-1.49	0.62	1.07	3.11E-01	Glycerophosphoinositols
871.657	7.56	M+K	$C_{55}H_{92}O_5$	DG 52:8	-0.70	0.54	1.00	4.01E-01	Diradylglycerols
975.6488	7.44	M+K	$C_{54}H_{97}O_{10}P$	PG 48:5	4.00	0.53	1.47	5.45E-01	Glycerophosphoglycerols
489.3432	5.76	M+Na	$C_{23}H_{51}N_2O_5P$	Sphinganine-1-phosphocholine	0.98	0.69	1.74	1.44E-02	Phosphosphingolipids
473.2009	0.7	M+H-2H$_2$O	$C_{22}H_{36}O_{13}$	Oleuropeoylsucrose	-1.63	0.63	1.16	1.67E-02	Saccharolipids
385.1118	3.34	M+H-H$_2$O	$C_{17}H_{22}O_{11}$	4-Hydroxy-5-(3',4'-dihydroxyphenyl)-valeric acid-O-glucuronide	-2.75	0.57	1.13	2.54E-01	Carbohydrates

Rt:Retention time.

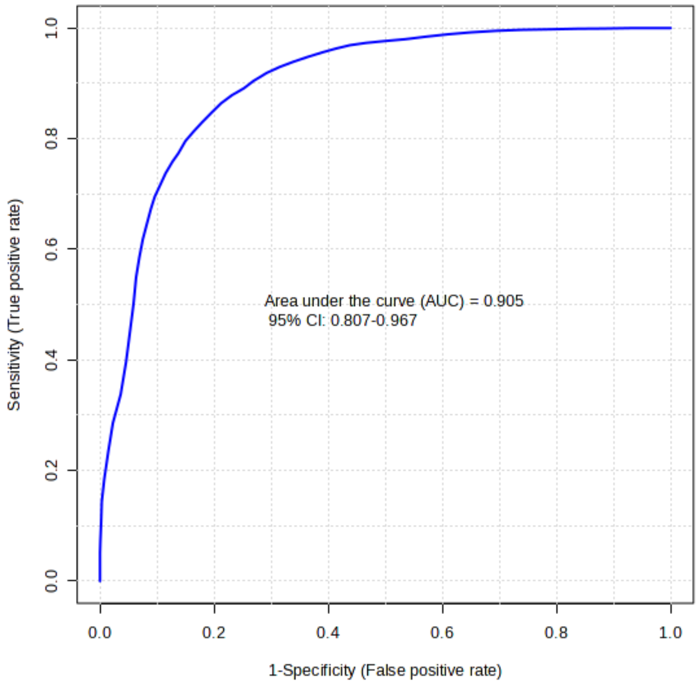

Figure S3. ROC curve for Model III, showing its average performance across all Monte Carlo cross-validation runs.

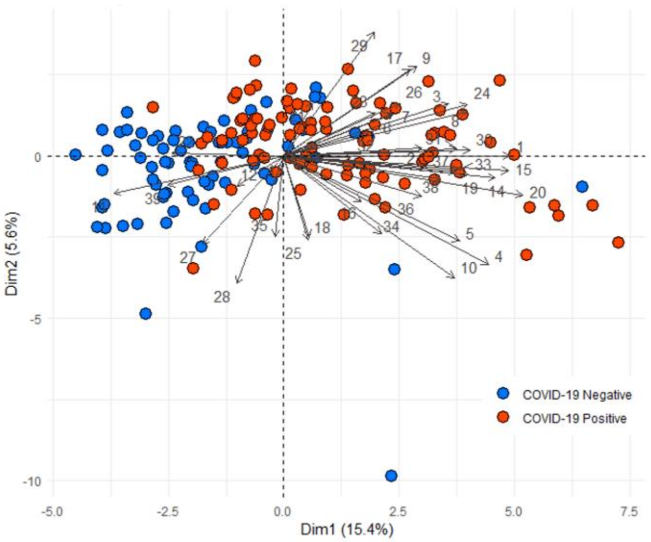

Figure S4. PCA biplot analysis of 39 metabolites identified in Model III. There are seven vectors (metabolites) in the negative quadrant of Component 1, where most of the COVID-19 negative samples are grouped. The positive quadrant of Component 1 contains the remaining metabolites (n=32), where most of the COVID-19-positive samples are grouped. The vector numbers represent the metabolites described in Table S4. Dim: dimension.

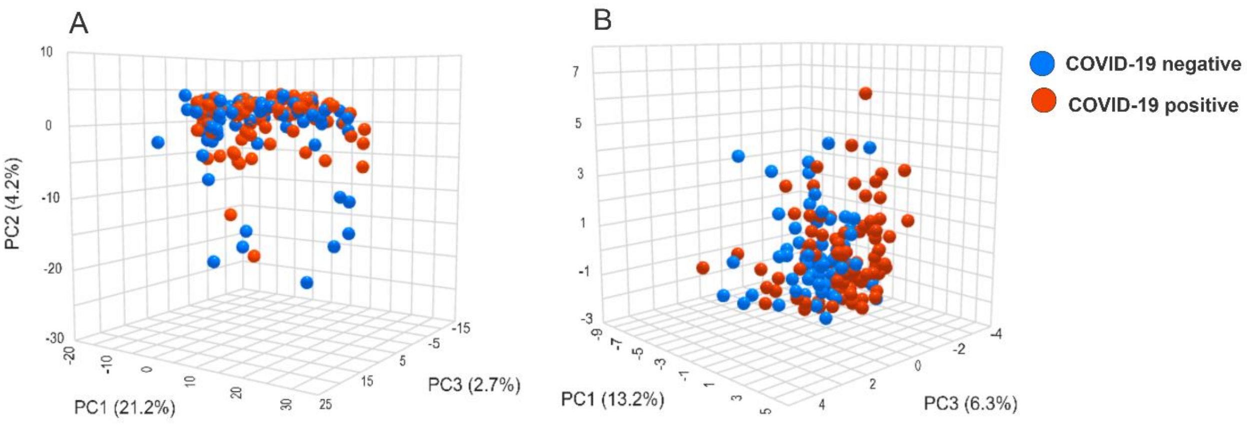

Figure S5. PCA score plots of the data detected in the positive ionization mode for COVID-19- positive and -negative groups. A) Three-dimensional principal component analysis (3D-PCA) score plots of all metabolites (n= 578) detected in the positive ion mode. B) 3D-PCA scores plot of 24 metabolites from Model IV. The orange dots represent the COVID-19-positive samples, and the blue dots represent the COVID-19-negative samples. PC: Principal component.

Table S6. Classification performance of Models III and IV using the Random Forest algorithm

	**Model III - Negative Mode** 39 metabolites (AUC 0.60-0.79)		**Model IV - Positive Mode** 24 metabolites (AUC 0.53-0.75)	
Algorithm	**Random Forest**		**Random Forest**	
Dataset (130/44)*	Training	Validation	Training	Validation
True Negative	45	16	39	11
False Positive	10	3	16	8
False Negative	11	0	19	6
True Positive	64	25	56	19
Sensitivity	85%	100%	75%	76%
Specificity	82%	84%	71%	57%
Bal. accuracy	84%	92%	73%	66%
PPV	86%	89%	78%	70%
NPV	80%	100%	67%	64%

* The training set was composed of 130 volunteers (55 COVID-19 negative and 75 COVID-19 positive), while the validation set consisted of 44 volunteers (19 COVID-19 negative and 25 COVID-19 positive). AUC: Area Under the Curve, PPV: Positive Predictive Value, NPV: Negative Predictive Value.
